# Clinical and Cost‐Effectiveness of Eye Movement Desensitisation and Reprocessing for Post‐Traumatic Stress Disorder in Children and Adolescents: A Systematic Review and Meta‐Analysis

**DOI:** 10.1002/cpp.70186

**Published:** 2025-12-04

**Authors:** Anthea Sutton, Christopher Carroll, Emma Simpson, Jessica Forsyth, Annabel Rayner, Shijie Ren, Matthew Franklin, Emily Wood

**Affiliations:** ^1^ Sheffield Centre for Health and Related Research, School of Medicine and Population Health University of Sheffield Sheffield UK

**Keywords:** adolescents, children, cost‐effectiveness, eye movement desensitisation and reprocessing, meta‐analysis, post‐traumatic stress disorder, systematic review

## Abstract

Eye movement desensitisation and reprocessing (EMDR) is a psychological therapy used to treat trauma. While trauma‐focused cognitive behavioural therapy (TF‐CBT) is often used, EMDR has potential for treating post‐traumatic stress disorder (PTSD). Previous research has focused on adult populations, with limited evidence for children and adolescents available. A systematic review was conducted to evaluate the clinical and cost‐effectiveness of EMDR for treating or preventing PTSD in children and adolescents. Randomised controlled trials (RCTs) were identified through a comprehensive search of six databases in September 2023. Eligibility criteria were based on the NICE 2018 PTSD guidelines. Data were extracted, and risk of bias was assessed using the Cochrane Risk of Bias 2.0 tool. Meta‐analyses were conducted where appropriate. Of 1220 unique records identified, nine studies met the inclusion criteria. Eight RCTs (*n* = 794 participants) explored clinical effectiveness, and one study examined cost‐effectiveness. Most studies compared EMDR with waitlist/usual care. A meta‐analysis demonstrated a significant and large effect size (SMD 1.57 95% CrI = 0.07–3.21) of EMDR treatment (delivered 3 months or more following trauma) compared with waitlist/usual care for children and adolescents with PTSD, in various populations including refugees, and victims of physical and/or sexual violence. Two trials compared EMDR with TF‐CBT and found no significant difference between therapies. From the very limited cost‐effectiveness evidence available, EMDR was ranked sixth out of 10 interventions. EMDR was demonstrated to be effective in reducing PTSD symptoms in children and adolescents, particularly when compared with waitlist/usual care. However, more high‐quality RCTs are needed to establish definitive conclusions. In addition, future research should prioritise within‐trial cost‐effectiveness analyses to provide a more comprehensive understanding of the cost–benefit profile of EMDR.

**Trial Registration:** PROSPERO prospective register of systematic reviews: CRD42023463360.

## Summary

- Eye movement desensitisation and reprocessing (EMDR) is an effective treatment for reducing PTSD symptoms in children and adolescents, with a large effect size observed when compared with waitlist or usual care.
- EMDR appears to be as effective as trauma‐focused cognitive behavioural therapy (TF‐CBT) in the child and adolescent population. This systematic review found no significant differences in clinical outcomes between the two therapies, suggesting that EMDR is a comparable and valid alternative.
- More high‐quality, methodologically sound studies are needed to provide definitive conclusions. Future research should prioritise well‐designed randomised controlled trials (RCTs) and include cost‐effectiveness analyses to provide a more complete picture of EMDR's benefits.

## 1 Introduction

Eye movement desensitisation and reprocessing (EMDR) is a psychological therapy for those experiencing trauma. While trauma‐focused cognitive behavioural therapy (TF‐CBT) remains the most widely used approach (Thielemann et al. 2022), EMDR has the potential to address the unique challenges of post‐traumatic stress disorder (PTSD) in younger populations (Hoppen, Meiser‐Stedman, et al. 2023; Adamowicz 2024).

Previous research has primarily focused on adult populations, exploring EMDR's effectiveness in various trauma contexts, such as combat, natural disasters and displacement (Kitchiner et al. 2019; Le Roux and Cobham 2022; Macgowan et al. 2022; Maglione et al. 2022). Systematic reviews and meta‐analyses have demonstrated comparable outcomes between EMDR and TF‐CBT in treating PTSD among adults (Hudays et al. 2022; Hoppen, Jehn, et al. 2023). However, the evidence base for children and adolescents is much less extensive, and there is a growing need for more comprehensive research on EMDR's efficacy for children and adolescents. In 2018, the UK National Institute for Health and Care Excellence (NICE) published guidance for recognising, assessing and treating PTSD in children, young people and adults (NICE 2018). The guidelines for children and adolescents were based on evidence reviews that identified literature up to January 2018 and included only two randomised controlled trials (RCTs) (Soberman et al. 2002; de Roos et al. 2017).

It is essential to conduct up‐to‐date systematic reviews to inform clinical guidelines and practice. This review aims to evaluate the most recent RCT evidence on EMDR's effectiveness, safety and cost‐effectiveness in treating or preventing PTSD in children and adolescents. By examining comparisons with alternative treatments or no treatment, this review will contribute to a more comprehensive understanding of EMDR's role in addressing the unique needs of young people affected by trauma.

The objective of this review was to update the rigorous NICE guidance and identify evidence published since 2018; therefore, the eligibility criteria mirrored those of the NICE 2018 guidelines (NICE 2018). In a recent survey of psychiatrists in 39 European countries, of those who used clinical guidelines, NICE was on a par with the World Health Organization (WHO) as the preferred source of guidelines (Rojnic Kuzman et al. 2024), demonstrating the relevance and use of updating the NICE review. Updating the evidence reviews from the NICE guidance is crucial for informing clinical practice and ensuring that therapists and their clients have access to the most up‐to‐date, evidence‐based treatment options. By evaluating recent research, this review directly informs therapists, including those using EMDR and other modalities, on the effectiveness of EMDR for children and adolescents with PTSD. This ensures that clinical decisions are guided by the best available evidence, ultimately benefiting patient care.

## 2 Materials and Methods

The systematic review was undertaken in accordance with the general principles recommended in the York CRD guidance (Akers et al. 2009) and the Preferred Reporting Items for Systematic Reviews and Meta‐Analyses (PRISMA) statement (Page et al. 2021) (see Appendix S1). The review protocol is registered on the PROSPERO prospective register of systematic reviews as CRD42023463360. The review question was: What is the clinical and cost‐effectiveness of EMDR for the prevention and treatment of children and adolescents with PTSD?

### 2.1 Searches

Systematic searches to identify RCTs and cost‐effectiveness studies were conducted in September 2023, using the following bibliographic databases: MEDLINE via Ovid, Embase via Ovid, PsycINFO via Ovid, Cochrane Library, CINAHL via EBSCO and PTSDpubs via ProQuest. The EMDR Publications Database maintained by the University of Sheffield for EMDR UK Members was also searched to cross‐check for any additional references. Searches were conducted using a combination of subject headings and free‐text search terms related to the population (people with PTSD) and the intervention (EMDR). The search strategy for MEDLINE can be found in Box 1. Boolean operators were used to combine these terms, and published methodological search filters were applied to identify RCTs, economic studies and systematic reviews (Glanville et al. 2025). A broad search including all ages was conducted, and results were separated into those relating to adults and those relating to children and adolescents. The review relating to adults is published separately (Simpson et al. 2025). The search strategy did not exclude any ages, and adolescents were classed as individuals aged from 10 to 19 years, as defined by the WHO (2025). When synthesising research on children and young people, chronological age is the most reliable variable for grouping and comparing participants across different studies in order to conduct reproducible systematic reviews. This is because standardised developmental stage measures are not consistently reported. All comparators eligible to be included in the review, where directly compared with EMDR; therefore, it was not necessary to include comparator terms in the search strategy. Searches were limited to studies published from 2018 onward to focus on evidence published since the NICE guidelines on PTSD. Pre‐2018 RCTs were sourced from the evidence review underpinning those guidelines. The search was not limited by language, but non‐English language studies and abstracts were excluded unless they provided sufficient information for data extraction and quality assessment.

**Box 1:**
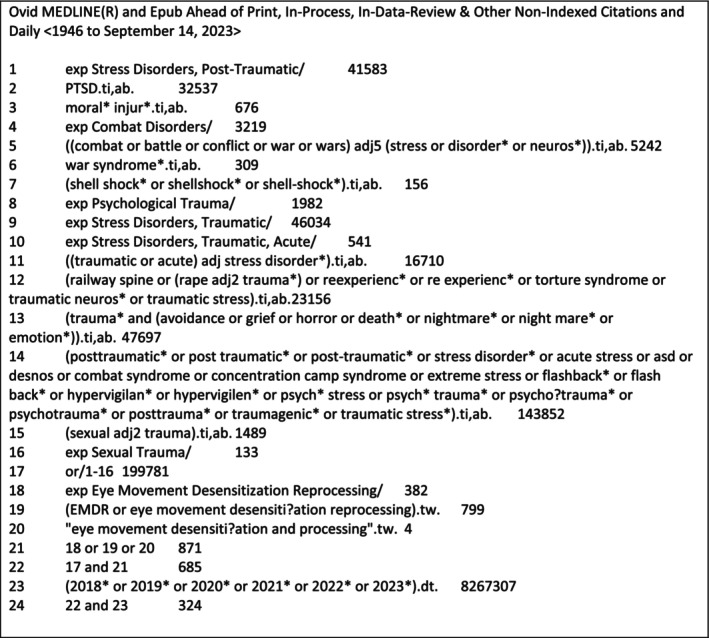
Search strategy for Ovid MEDLINE.

The search strategy was developed on MEDLINE via Ovid, with input from clinical experts and was peer‐reviewed by a second information specialist using the PRESS checklist (McGowan et al. 2016). Supplementary searching included reference list screening of included studies and relevant systematic reviews and hand searching of key journals and websites. An update search of the EMDR Publications Database was conducted in January 2025 to check for potentially relevant studies since the original literature search was conducted. The full search strategies and sources can be found in Appendix S2.

### 2.2 Study Selection, Data Extraction and Risk of Bias Assessment

As this review aimed to update the findings reported in the 2018 NICE guidelines (NICE 2018), the eligibility criteria applied were consistent with those used in the original guidelines (see Table 1). Therefore, studies in specific populations with the coexisting conditions, adjustment disorders, traumatic grief, psychosis and learning disabilities, were excluded. Reported symptoms relating to comorbidities such as anxiety and depression were analysed as secondary outcomes, recognising that PTSD often involves significant psychiatric comorbidity, particularly high rates of co‐occurring depression and anxiety symptoms (Qassem et al. 2021), the reduction of which is relevant for assessing overall treatment success.

**TABLE 1:** Eligibility criteria.

Study design	For clinical effectiveness and safety, RCTs only; for cost‐effectiveness studies, the outcome is quality‐adjusted life years (QALYs).
Participants/population	Treatment: Children and adolescents with PTSD (as defined by a diagnosis of PTSD according to Diagnostic and Statistical Manual of Mental Disorders [DSM], International Classification of Diseases [ICD] or similar criteria). Prevention: (a) within the first month after an event or events, (b) ongoing exposure to trauma (e.g., in a war zone) or (c) children and adolescents and young people with subthreshold symptoms of PTSD, after at least 1 month.
Intervention	Eye movement desensitisation and reprocessing (EMDR).
Comparators	Any psychological trauma‐focused cognitive behavioural therapy (TF‐CBT), psychosocial therapy or nonpharmacological therapy; waitlist; and care as usual.
Primary outcomes	PTSD symptoms/response/remission/relapse and QALYs.
Additional outcomes	Discontinuation for any reason (a proxy for acceptability of the intervention); dissociative symptoms; personal/social/occupational functioning (including global functioning/functional impairment); sleeping difficulties; quality of life; symptoms of a coexisting condition (including anxiety, depression and substance misuse problems); safety/adverse events (AEs); and treatment duration, patient time engaged with treatment.
Publication date	2018 onwards (earlier RCT evidence was sourced from the comprehensive 2018 NICE evidence review for their guideline on PTSD [ref]).
Exclusion	All other study designs. RCTs with fewer than *n* = 10 participants. Editorials, book chapters and conference papers and dissertations. Populations with adjustment disorders, traumatic grief, psychosis as a coexisting condition and learning disabilities. Studies of adults (a systematic review of the evidence in adults is the subject of a separate publication).

Abbreviations: EMDR, eye movement desensitisation and reprocessing; PTSD, post‐traumatic stress disorder; RCT, randomised controlled trial.

Identified records were imported into Covidence software (Veritas Health Innovation, n.d.). Two reviewers independently conducted study selection at both the title/abstract and full‐text stages, using the eligibility criteria outlined in Table 1. Disagreements between reviewers were resolved through consensus or by consulting a subject expert. For clinical effectiveness and safety, data were extracted into a prepiloted data extraction form by one reviewer and checked by a second reviewer. Disagreements were resolved through consensus or by consulting a subject expert. The following data were tabulated: study characteristics, participant characteristics, intervention and comparator details and clinical outcome measures and results. As per the NICE 2018 guidelines, we searched for and extracted quantitative data only, outcomes that are formally measured using validated scales.

For cost‐effectiveness studies, data were collected on the following study characteristics:

- Publication information (author, year and journal)
- Study design (country, population, perspective [outcome and costs], analysis type [within‐trial and statistical methods used or modelling/modelling‐type], outcome measure and associated detail [e.g., preference‐based measure and utility value set], time horizon, comparators, intervention duration, cost type, discount rates and year of valuation)
- Study outcomes (results [quality‐adjusted life years {QALYs}/costs, incremental QALYs/incremental costs, incremental cost‐effectiveness ratios (ICERs), probability of cost‐effectiveness] and sensitivity analysis)

For RCTs, the quality of included studies was assessed using the validated Cochrane Risk of Bias 2.0 tool (Sterne et al. 2019). This assessment focused on the primary outcome of our review and was conducted by one reviewer, with a second reviewer checking for consistency. Disagreements were resolved through consensus or by consulting a third reviewer if necessary. All processes outlined above were applied to the trials identified from both the update searches and the NICE evidence reviews.

### 2.3 Methods of Data Synthesis for Clinical Effectiveness

A minimum of three studies were required for statistical assessment via pairwise meta‐analysis (Dias et al. 2013). To be included in a pairwise meta‐analysis, a study had to provide both mean and standard deviation (SD) for changes in PTSD symptoms from pretreatment to post‐treatment, or these data had to be calculable.

The primary outcome of interest was the change in PTSD symptoms, expressed as a standardised mean difference (SMD) to facilitate comparisons across studies using different scoring methods. Detailed information regarding the assumptions, calculations and statistical analyses used to assess treatment effects can be found in the Supporting Information. A positive change in SMD indicated improvement, while a negative change indicated worsening of symptoms. Only one meta‐analysis was possible, focusing on delayed treatment of children and adolescents with PTSD (i.e., 3 months or more following trauma). Meta‐analyses for prevention or early treatment were not feasible due to insufficient data.

Because data were selected from studies from independent researchers, a common effect size could not be assumed. Therefore, a random‐effects model was used to account for heterogeneity in treatment effects. Model parameters were estimated using a Bayesian framework, with details provided in Appendix S3. All analyses were conducted using the freely available software WinBUGS (Lunn et al. 2000) via the R package, R2WinBUGS (Sturtz et al. 2005). Results are presented alongside the posterior median treatment effects and 95% credible intervals (CrI). Effect sizes were graded using Cohen's criteria: not substantial (SMD < 0.2), small (0.2 ≤ SMD < 0.5), medium (0.5 ≤ SMD < 0.8) and large (0.8 ≤ SMD) (Cohen 2013). Study heterogeneity was assessed and interpreted using established categories (Ren et al. 2018).

For comparisons where meta‐analysis was not feasible, for example, where the required minimum of three studies was not met, or data were not provided in a suitable format for statistical calculation, a narrative synthesis was conducted. Narrative synthesis was also used to report on all secondary outcomes. For the narrative synthesis, data were systematically grouped into tables to analyse.

## 3 Results

### 3.1 Search Results

**FIGURE 1:**
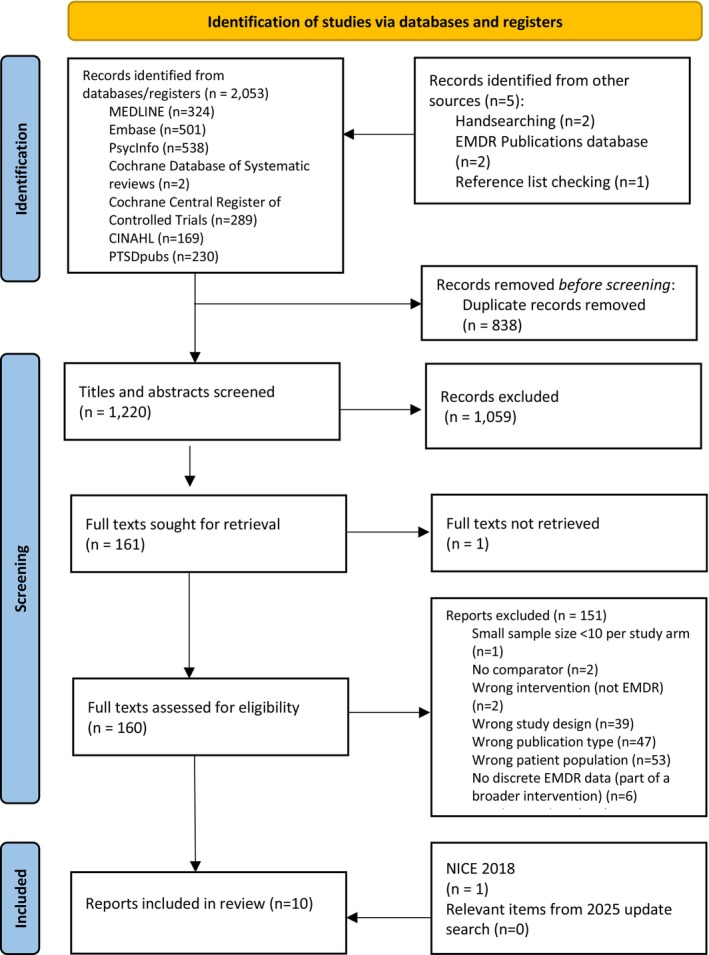
Flow diagram. *Source:* Page et al. (2021).

More than 1200 titles and abstracts were screened from the bibliographic database searches, 160 full‐text articles were checked and eight studies (nine reports) were found that met the inclusion criteria (Figure 1). One study focused on cost‐effectiveness, while the remaining seven studies (eight reports) reported clinical effectiveness data for PTSD outcomes (Figure 1). An additional study was identified from the 2018 NICE guidelines (de Roos et al. 2017). This was the only relevant study from the original 2018 NICE review. One other trial evaluating EMDR in children or adolescents with PTSD was included in the NICE 2018 guidance (Soberman et al. 2002) but was excluded from this review because only 31% of participants had a diagnosis of PTSD and discrete data for this group were not reported. The primary diagnosis for most participants in the study was conduct disorder (59%). The update search in January 2025 identified 16 potential new studies, but none satisfied the inclusion criteria. Therefore, this review includes a total of eight relevant RCTs evaluating EMDR in children and adolescents with PTSD from both before and after 2018 and represents the total evidence of relevant studies satisfying the inclusion criteria.

### 3.2 Clinical Effectiveness Results

Eight RCTs (nine reports) published between 2017 and 2021 were identified providing up‐to‐date data on EMDR's clinical effectiveness for PTSD in children and adolescents (de Roos et al. 2017; Osorio et al. 2018; Jaberghaderi et al. 2019; Molero et al. 2019; Jiménez et al. 2020; Meentken et al. 2020, 2021; Karadag et al. 2021; Banoglu and Korkmazlar 2022). Tables 2 and 3 provide details of the study and participant characteristics of these trials.

**TABLE 2:** Characteristics of trials comparing EMDR with CBT and/or non‐CBT active or passive interventions for treatment or prevention or treatment of PTSD.

Author, year	Location (country)	Design	Number randomised	Intervention (EMDR)		Comparator(s)		Treatment duration	Follow‐up	PTSD outcome measures
				*N* ^a^	Details	*N* ^a^	Details			
Delayed, treatment										
Banoglu, 2021	Turkey	Single‐centre, open‐label RCT	61	42	EMDR group protocol with children (EMDR‐GP/C): Each research participant joined three to four group sessions until they reported a SUD rating of 0; every group session had eight participants on average. An EMDR‐GP/C session took 90–120 min.	19	WL	Unclear	Unclear (post‐treatment)	Self‐report CTPS‐RI
de Roos, 2017	Netherlands	Multicentre single‐blind, open‐label RCT	103	43	EMDR: Standard eight‐phase protocol. Up to six weekly, individual sessions lasting up to 45 min.	42	TF‐CBT (narrative exposure therapy [NET], that is, CBWT)	6 weeks	12 months	Self‐report CTRI
						18	WL			
Jaberghaderi, 2019	Iran	Single‐centre, open‐label RCT	139	40	EMDR: Standard eight‐phase protocol. At least three sessions with age‐appropriate modifications; duration of the sessions was 45 min and some took the full 60 min.	40	CBT: 12 sessions; based on Kolko and Swenson's protocol of abuse‐focused CBT.	Unclear: Termination criteria were a max. of 12 sessions of any treatment (min. of 6 for CBT)	2 weeks (post‐treatment)	Self‐report CROPS/PROPS
						59	WL			
Jimenez, 2020	Mexico	Multicentre, open‐label RCT	32	16	EMDR‐PRECI: eight‐phase and three‐pronged protocol of 60‐min sessions provided two to three times a week depending on availability of participants.	16	TAU^b^	EMDR‐PRECI: Average of 4.68 individual sessions; TAU: Average of 12.6 individual sessions	90 days	Self‐report PCL‐5
Karadag, 2021	Turkey	Community‐based RCT	178	88	EMDR‐Derived Self‐Help Psychological Crisis Intervention Guide (PDF document sent to children). Intervention group carried out the guide's activities three times in total, once every 2 days, at home with the parents' assistance. Each session took an average of 20 min.	90	WL (4 weeks)	1 week	4 weeks	Self‐report CTPS‐RI
Molero, 2019	Spain	Multicentre, open‐label RCT	184	93	EMDR‐IGTP‐OTS: participants received an average of 8 h of treatment over nine group treatment sessions; treatment focused only on the distressing memories related to their life as refugees; included butterfly hug (BH) as a self‐administered bilateral stimulation method to process traumatic material.	91	None	Three days (nine sessions [first session average 95 min, other sessions average 48 min]; three times a day during three consecutive days)	90 days	Self‐report PCL‐5
Delayed, prevention^a^										
Meentken 2020, 2021	Netherlands	Single‐centre, open‐label RCT	74	37	EMDR: Standard Dutch EMDR protocol for children and adolescents; sessions of approximately 50 min. Treatment was completed when (1) SUDs of all selected memories regarding the medical trauma were zero and/or (2) positive cognitions were established (rated by the child) and/or (3) child, parents and therapist agreed that PTSD symptoms had sufficiently decreased.	37	CAU	Mean = 3.5 (SD = 1.9) sessions	Mean = 9.7 (SD = 2.5) weeks after the first EMDR session	Self‐report CRTI
Osorio, 2018	Mexico	Single‐centre, open‐label RCT	23	12	EMDR‐IGTP‐OTS: Participants received an average of 8 h of treatment over nine group treatment sessions; treatment focused only on the distressing memories related to cancer.	11	None	Two days (six sessions [first session average 106 min, other sessions average 53 min]; three times a day during two consecutive days)	90 days	Self‐report PCL‐5

Abbreviations: CAU, care as usual; CBWT, cognitive behavioural writing therapy; CROPS, Child Report of Post‐Traumatic Stress Symptoms (Persian version); CRTI, Children's Responses to Trauma Inventory; EMDR, eye movement and desensitisation reprocessing; GP/C, group protocol with children; IGTP‐OTS, Interactive Group Treatment Protocol—Ongoing Traumatic Stress; PCL‐5, DSM‐5 Post‐Traumatic Checklist; PRECI, Protocol for Recent Critical Incidents; PROPS, Parent Report of Post‐Traumatic Stress Symptoms (Persian version); PTSD, post‐traumatic stress disorder; SUD, subjective units of distress; TF‐CBT, trauma‐focused CBT; WL, waitlist.

^a^ Based on participants having subthreshold PTSD scores at baseline.

^b^ Sixty‐minute sessions of psychological support, oriented to life plan and emotions management provided once a week.

**TABLE 3:** Participant characteristics of trials comparing EMDR with CBT and/or non‐CBT active or passive interventions for treatment or prevention or treatment of PTSD.

Author, year	Intervention	Single/multiple/mixed traumatic events	Type of trauma	Time since traumatic event in months (mean)	Time since PTSD onset in months (mean)	Age in years (mean)	Sex: female (%)	Ethnicity *N* (%)
Delayed, treatment								
Banoglu, 2021	EMDR‐GC/P	Unclear (probably multiple)	Syrian refugees; war‐related trauma	> 6 months	NR	NR	Overall: 41	NR
	WL		Syrian refugees; war‐related trauma	> 6 months	NR	NR	Overall: 41	NR
de Roos, 2017	EMDR	Single	Mixed—Physical abuse/assault (23%); sexual abuse (26%); accident/injury of a loved one (19%); traumatic loss (18%); disaster/other (13%)	18.30	NR	12.96	53.5	NR
	CBWT			16.26	NR	13.41	59.5	NR
	WL			13.00	NR	12.47	61.1	NR
Jaberghaderi, 2019	EMDR	Unclear (probably multiple)	CPA: 3 (8%); PC: 8 (20%); CPA and PC 29 (72%)	NR	NR	8–9: 8.3%; 9.5–10.5: 37.5%; 11–12: 54.2%	50	NR
	CBT		CPA: 10 (24%); PC: 0 (0%); CPA and PC 30 (76%)	NR	NR	8–9: 12%; 9.5–10.5: 36%; 11–12: 52%	48	NR
	WL		CPA: 30 (51%); PC: 14 (24%); CPA and PC 15 (25%)	NR	NR	8–9: 9.4%; 9.5–10.5: 43.4%; 11–12: 47.2%	51	NR
Jimenez, 2020	EMDR‐PRECI	Unclear	Female victims of sexual and/or physical violence	NR	NR	Overall: 15.35	100	NR
	TAU^b^			NR	NR	Overall: 15.35	100	NR
Karadag, 2021	EMDR	Single	COVID‐19 pandemic	NR	NR	Overall: 9.07	Overall: 55.1	NR
	WL			NR	NR	Overall: 9.07	Overall: 55.1	NR
Molero, 2019	EMDR‐IGTP‐OTS	Single	Refugees	NR	NR	Overall: 16.36	0	NR
	No treatment			NR	NR	Overall: 16.36	0	NR
Delayed, prevention^a^								
Meentken 2020, 2021	EMDR	Mixed: Single: 9 (24.3%) Multiple: 28 (75.7%)	COVID‐19 pandemic: Hospitalisation of at least one night	Time since last medical event in years 1.7 ± 1.5	NR	9.8	32.4	Dutch 32 (88.9%); other Western 2 (5.6%); non‐Western 2 (5.6%)
	CAU	Mixed: Single: 7 (18.9%) Multiple: 30 (81.1%)		Time since last medical event in years 1.8 ± 1.4	NR	9.4	35.1	Dutch 27 (75%); other Western 2 (5.6%); non‐Western 7 (19.4%)
Osorio, 2018	EMDR‐IGTP‐OTS	Single	Cancer	Overall range: < 1 year to > 10 years	NR	Overall: 16.71	Overall: 43.5	NR
	No treatment			Overall range: < 1 year to > 10 years	NR	Overall: 16.71	Overall: 43.5	NR

Abbreviations: CAU, care as usual; CBT, cognitive behavioural therapy; CPA, child physical abuse; EMDR, eye movement and desensitisation reprocessing; NR, not reported; PC, parental conflicts; WL, waitlist.

^a^ Based on participants having subthreshold PTSD scores at baseline.

^b^ TAU: psychological support, oriented to life plan and emotions management.

All studies were unblinded; four studies were based in a single centre (Osorio et al. 2018; Jaberghaderi et al. 2019; Meentken et al. 2020, 2021; Banoglu and Korkmazlar 2022), three were multicentre (de Roos et al. 2017; Molero et al. 2019; Jiménez et al. 2020) and one was community based (Karadag et al. 2021). This latter study (Karadag et al. 2021) evaluated a unique self‐help EMDR intervention, and as such represented a very different approach to EMDR compared with all other studies. The studies were conducted in the Netherlands (*n* = 2) (de Roos et al. 2017; Meentken et al. 2020, 2021), Mexico (*n* = 2) (Osorio et al. 2018; Jiménez et al. 2020), Turkey (*n* = 2) (Karadag et al. 2021; Banoglu and Korkmazlar 2022), Spain (Molero et al. 2019) and Iran (Jaberghaderi et al. 2019). Data were collected at multiple timepoints in studies, from immediate post‐treatment assessments to a maximum follow‐up; these follow‐up periods ranged from 2 weeks (Jaberghaderi et al. 2019) to 1 year (de Roos et al. 2017); the follow‐up duration was unclear in one study (Banoglu and Korkmazlar 2022).

The trials evaluated a range of EMDR delivery approaches: Three studies delivered an EMDR group protocol (Osorio et al. 2018; Molero et al. 2019; Banoglu and Korkmazlar 2022), two for ongoing traumatic stress (OTS) (Osorio et al. 2018; Molero et al. 2019). One study delivered the Protocol for Recent Critical Incidents (PRECI) protocol (Jiménez et al. 2020). Group therapy interventions tended to be more intensive. The number of treatment sessions differed across the studies, where reported, from as few as a mean of 3.5 sessions (Meentken et al. 2020, 2021) to a maximum of 12 sessions (Jaberghaderi et al. 2019), over differing periods of time, from 2 days (with six sessions) (Osorio et al. 2018) to 1 week (Karadag et al. 2021) or several weeks (de Roos et al. 2017; Jiménez et al. 2020).

All studies compared EMDR with waitlist/treatment as usual/no treatment. Two studies also included a third arm: both compared EMDR with TF‐CBT, with treatments lasting 4–12 (Jaberghaderi et al. 2019) or 6 weeks (de Roos et al. 2017). Most studies assessed the effectiveness of delayed treatment (delivered 3 months or more following trauma); only two studies examined EMDR for prevention of PTSD (children and adolescents had subclinical levels of PTSD) (Osorio et al. 2018; Meentken et al. 2020, 2021). All trials used self‐report measures only for children and/or parents, three studies used the DSM‐5 Post‐Traumatic Checklist (PCL‐5) (Osorio et al. 2018; Molero et al. 2019; Jiménez et al. 2020), two studies used Childhood Post‐Traumatic Stress Reaction Index (CTPS‐RI) (Karadag et al. 2021; Banoglu and Korkmazlar 2022), two studies used the Children's Responses to Trauma Inventory (CRTI) (de Roos et al. 2017; Meentken et al. 2020, 2021) and one study used Child/Parent Report of Post‐Traumatic Stress Symptoms (CROPS/PROPS), Persian version (Jaberghaderi et al. 2019).

The populations studied (see Table 3) included refugees (Molero et al. 2019; Banoglu and Korkmazlar 2022), childhood abuse and/or parental conflict (Jaberghaderi et al. 2019), female victims of sexual and/or physical violence (Jiménez et al. 2020), individuals hospitalised with or affected by COVID‐19 (Meentken et al. 2020, 2021; Karadag et al. 2021) and cancer patients (Osorio et al. 2018). Children and adolescents in one study had been exposed to a wide range of different traumas, including physical and sexual abuse and disasters (de Roos et al. 2017). Four studies included populations with PTSD following a single traumatic event (de Roos et al. 2017; Osorio et al. 2018; Molero et al. 2019; Karadag et al. 2021), and one study was in a mixed population (single and multiple trauma) (Meentken et al. 2020, 2021). The remaining four studies did not clearly specify whether participants had experienced single, multiple or ongoing trauma.

According to the WHO definition, adolescents are between 10 and 19 years old (WHO 2025). All studies included adolescent participants (see Table 3), with some studies potentially only including this age group (data unclear). Two studies reported a mean age of between 9 and 10 years (Meentken et al. 2020, 2021; Karadag et al. 2021), one study reported that participants fell within an age range of 8–12 years (Jaberghaderi et al. 2019), one study reported a mean age of approximately 13 years (de Roos et al. 2017) and three studies reported a mean age of between 15 and 17 years (Osorio et al. 2018; Molero et al. 2019; Jiménez et al. 2020). One study did not report a mean age, only a range of 6–15 years as an inclusion criterion (Banoglu and Korkmazlar 2022). The female:male split was generally even in most studies, but one study was conducted exclusively with female children and adolescents (female victims of physical or sexual violence) (Jiménez et al. 2020) and one exclusively with male refugees (Molero et al. 2019).

### 3.3 Risk of Bias Assessments

**FIGURE 2:**
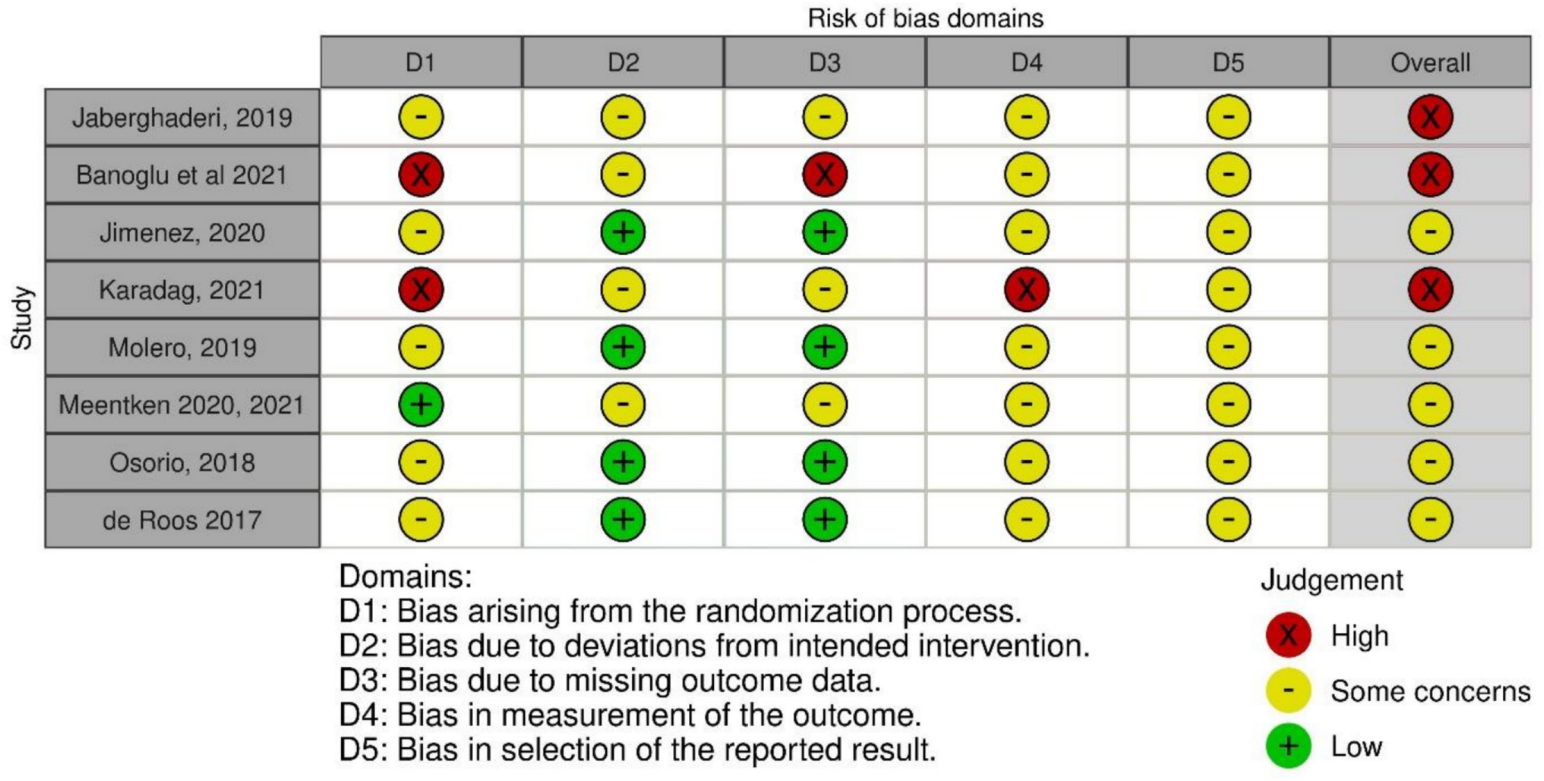
Cochrane Risk of Bias assessment of included studies.

All of the trials conducted in children and adolescents were assessed as being at either high (de Roos et al. 2017; Banoglu and Korkmazlar 2022; Jaberghaderi et al. 2019; Karadag et al. 2021) or moderate risk of bias (Jiménez et al. 2020; Molero et al. 2019; Meentken et al. 2020; Osorio et al. 2018) (see Figure 2). The primary methodological concerns identified in the trials were related to randomisation, outcome measurement and selective reporting. While randomisation was attempted, inadequate allocation concealment may have led to imbalances between groups. The unblinded nature of the trials increased the risk of observer bias in primary and secondary outcome measurement, with rare exceptions for any outcome measure (de Roos et al. 2017). Additionally, the lack of detailed trial protocols raised concerns about potential selective reporting of outcomes. Generally, there were no reported deviations from the planned interventions, and missing data were not a significant issue due to the short duration of treatment and follow‐up.

### 3.4 PTSD Results

To make the comparisons more homogenous and robust, and in accordance with the previous NICE guidelines (NICE 2018), the studies were grouped and analysed based on the following criteria:

- Comparator: TF‐CBT or waitlist/usual care/no treatment
- Treatment timing: Delayed (more than 3 months post‐trauma) or early (within 3 months)
- Follow‐up: Post‐treatment or later duration
- Outcome assessment: Self‐report or clinician assessed

All of the included studies assessed self‐report only, and therapy more than 3 months after the index event (delayed). The PTSD results for all included studies are presented in Table 4. A total of 794 patients contributed PTSD data, with *n* = 371 randomly assigned to EMDR, *n* = 82 to TF‐CBT and *n* = 341 to waitlist/usual care/no treatment. The studies were heterogeneous in terms of populations and comparisons.

**TABLE 4:** Results of trials comparing EMDR with CBT and/or non‐CBT active or passive interventions for treatment or prevention or treatment of PTSD.

Author, year	Comparator	PTSD self‐report scale used	Follow‐up	EMDR mean change from baseline (SD)	Comparator mean change from baseline (SD)	EMDR versus comparator treatment group × time interaction
Delayed, treatment						
Banoglu, 2021	WL	CPTS‐RI	Post‐treatment	12.22 (11.27)	4.92 (9.77)	*p* = 0.036
de Roos, 2017	TF‐CBT	CTRI	Post‐treatment	32.24	34.30	Overall, *p* = 0.60
			3 months	31.31	36.63	
			12 months	35.81	39.40	
	WL		Post‐treatment	32.24	6.02	*p* < 0.001
Jaberghaderi, 2019	TF‐CBT	CROPS	2 weeks	9.45 (9.82)	10.36 (7.60)	*p* = 0.658
	WL				0.25 (10.70)	*p* = 0.004
Jimenez, 2020	Treatment as usual	PCL‐5	Post‐treatment	34.4 (47.54)	1.25 (7.07)	*p* < 0.001
			90 days	37.7 (54.64)	−0.67 (worsens) (7.10)	*p* < 0.001
Karadag, 2021	WL	CPTS‐RI	Post‐treatment	Mean NR, median 7.5	Mean NR, median 6.8	*p* = 0.006
Molero, 2019	No treatment	PCL‐5	Post‐treatment	18.53 (18.46)	4.64 (15.43)	*p* < 0.001
			90 days	25.70 (14.00)	8.97 (13.85)	*p* < 0.001
Delayed, prevention						
Meentken, 2020, 2021	Care as usual	CTRI	8 weeks (mean 9.75 weeks)	Child: 13.00 (11.69); parent: 11.57 (11.64)	Child: 12.83 (11.37); parent: 8.03 (12.47)	NR
			8 months	Child: 15.03 (11.11); parent: 15.14 (10.97)	Child: 13.60 (10.68); parent: 11.27 (11.29)	NR
Osorio, 2018	No treatment	PCL‐5	Post‐treatment	12.19 (29.17)	−0.33 (10.92)	*p* < 0.01
			90 days	16.37 (10.91)	−1.25 (10.99)	*p* < 0.01

Abbreviations: CBT, cognitive behavioural therapy; CPTS‐RI, Childhood Post‐Traumatic Stress Reaction Index; CROPS, Child Report of Post‐Traumatic Stress Symptoms (Persian version); EMDR, eye movement and desensitisation reprocessing; NR, not reported; PCL‐5, DSM‐5 Post‐Traumatic Checklist; PTSD, post‐traumatic stress disorder; SD, standard Deviation; TF, trauma focused; WL, waitlist.

#### 3.4.1 EMDR vs. TF‐CBT for Treatment of PTSD

Two studies (de Roos et al. 2017; Jaberghaderi et al. 2019) compared delayed (three or more months after trauma) EMDR with TF‐CBT for treating PTSD in children and adolescents. Both treatments led to improvements, but there were no significant differences between the groups at follow‐ups of 2 weeks (Jaberghaderi et al. 2019), 3 months or 12 months (de Roos et al. 2017). EMDR sessions were conducted at least three times, while a maximum of 12 TF‐CBT sessions were delivered. Due to the limited number of studies (< 3), a meta‐analysis was not possible for this comparison.

#### 3.4.2 EMDR vs. Waitlist/Usual Care/No Treatment for Treatment of PTSD

Six studies (de Roos et al. 2017; Jaberghaderi et al. 2019; Molero et al. 2019; Jiménez et al. 2020; Karadag et al. 2021; Banoglu and Korkmazlar 2022) compared EMDR with waitlist or usual care for children and adolescents with PTSD. All studies showed improvement in the EMDR group, and EMDR was significantly better than the control conditions. One study reported a decline in the usual care group at the 90‐day follow‐up (Jiménez et al. 2020).

**FIGURE 3:**
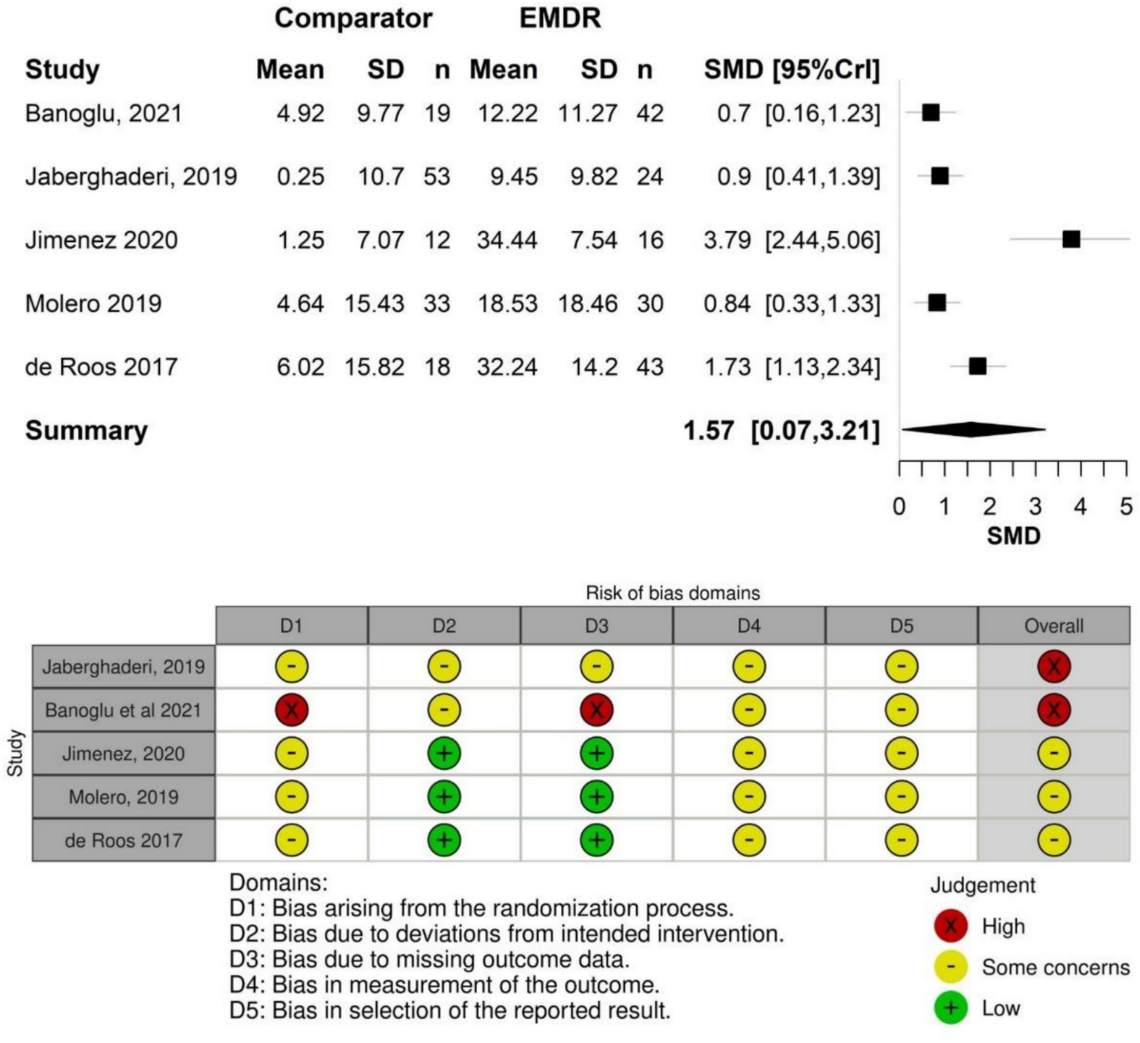
Meta‐analysis for delayed EMDR treatment in children and adolescents with PTSD versus waitlist/usual care/no treatment (self‐reported outcomes) and risk of bias assessment.

Five of the six studies were suitable for meta‐analysis. The Karadag trial (Karadag et al. 2021) did not provide data in a suitable format for meta‐analysis and involved a unique self‐help intervention, making it less comparable with traditional EMDR therapy. Two of the studies were considered to have a high risk of bias (Banoglu and Korkmazlar 2022; Jaberghaderi et al. 2019), while the remaining three were at moderate risk (de Roos et al. 2017; Molero et al. 2019; Jiménez et al. 2020). A total of 393 participants were included across the four studies. A pairwise meta‐analysis was conducted to evaluate the overall effect of EMDR compared with the control conditions of waitlist/usual care. Figure 3 presents the SMD of EMDR compared with waitlist/usual care, as measured by self‐reported scores in children and adolescents.

The meta‐analysis found that EMDR was significantly more effective than waitlist, usual care or no treatment for children and adolescents with PTSD, with a large effect size. The SMD was 1.57 (95% CrI = 0.07–3.21). However, there was substantial heterogeneity in the results between studies, as indicated by a between‐study SD of 1.55 (95% CrI = 0.69–2.65). According to the interpretation presented by Ren et al. (2018), this suggests that the treatment effect in one study could be up to 50 times larger than that in another study; this is likely to be due to the much larger treatment effect observed in the Jimenez 2020 study relative to the other four studies, as well as other potential differences in study design and populations.

The previous NICE review (NICE 2018) found no significant difference between EMDR and waitlist/usual care, though the results slightly favoured EMDR (SMD 0.90, 95% CrI −0.85 to 2.64). That finding was based on only two studies (de Roos et al. 2017; Soberman et al. 2002), one of which (Soberman et al. 2002) only included a small proportion of trial participants with PTSD (31%), and no discrete outcome data for this subgroup, and so was excluded from the current review.

#### 3.4.3 EMDR vs. Waitlist/Usual Care/No Treatment for Prevention of PTSD

Two studies compared EMDR with usual care/no treatment for prevention of PTSD in children and adolescents and reported improvements in the EMDR group (Osorio et al. 2018; Meentken et al. 2020, 2021). However, only one study directly compared the two groups and found a statistically significant advantage for EMDR (Osorio et al. 2018). Due to the limited number of studies (< 3), and the lack of SD data in one study, a meta‐analysis was not possible for this comparison.

### 3.5 Other Outcomes

A range of other outcomes was also reported by these trials, including discontinuation and adverse event rates, depression, anxiety and quality of life scores (data provided in Appendix S4).

Six of the seven included studies reported discontinuation rates (Osorio et al. 2018; Jaberghaderi et al. 2019; Molero et al. 2019; Jiménez et al. 2020; Karadag et al. 2021; Meentken et al. 2021; Banoglu and Korkmazlar 2022). There were no significant differences in discontinuation rates between EMDR and TF‐CBT. When comparing EMDR with waitlist/usual care, the evidence was inconsistent. Some studies reported lower discontinuation rates with EMDR (Jiménez et al. 2020; Karadag et al. 2021; Banoglu and Korkmazlar 2022), while others reported higher rates (Jaberghaderi et al. 2019). However, the studies varied in terms of participant, intervention and comparator details. Some settings, such as those with displaced populations (Molero et al. 2019; Banoglu and Korkmazlar 2022), were less conducive to treatment continuation due to external factors.

Three of the seven included studies reported on adverse events (Osorio et al. 2018; Molero et al. 2019; Jiménez et al. 2020) and found no significant adverse effects associated with EMDR or the comparator, treatment as usual (Jiménez et al. 2020).

Six studies assessed depression and/or anxiety outcomes in children and adolescents (Osorio et al. 2018; Molero et al. 2019; Jiménez et al. 2020; Meentken et al. 2020, 2021; Karadag et al. 2021; Banoglu and Korkmazlar 2022). Given the high rates of comorbidity with PTSD, these outcomes help to measure the holistic effectiveness of treatments. Studies comparing EMDR with waitlist/usual care or no treatment generally found significant reductions in depression and anxiety symptoms following EMDR treatment relative to the comparator. However, while the data suggest that EMDR may have a positive impact on both depression and anxiety, this evidence is limited by the small number of studies, methodological limitations and variability between studies. All of the studies contributing data on these outcomes were at high or moderate risk of bias; had relatively small sample sizes; and demonstrated some heterogeneity in terms of details of populations, interventions and comparators.

### 3.6 Cost‐Effectiveness Results

None of the RCTs included in the clinical effectiveness section provided a within‐trial cost‐effectiveness analysis, which would have given insight into the cost of delivering the intervention, alongside the causal impact of future costs and general health outcomes. One modelling‐based cost‐effectiveness study was identified by the search (Mavranezouli, Megnin‐Viggars, Grey, et al. 2020). The effectiveness evidence was based on a network meta‐analysis that included 29 RCTs for changes in PTSD symptom scores between baseline and treatment endpoint and 10 RCTs for changes in PTSD symptom scores between baseline and 1‐ to 4‐month follow‐up (Mavranezouli, Megnin‐Viggars, Trickey, et al. 2020).

The model used a hybrid decision‐analytic model of a decision tree followed by a Markov model. The cohort was based on those under the age of 18. The population concerned those with clinically important PTSD with symptoms present for more than 3 months after the incident. Cognitive therapy, a form of TF‐CBT, was the most cost‐effective intervention, while EMDR was the sixth most cost‐effective intervention, out of 10 interventions and no treatment. However, this ranking separates TF‐CBT into multiple subtypes (cognitive, narrative exposure, exposure/PE, Cohen TF‐CBT/CPT and group therapy), with all but one ranking higher than EMDR. When considering these TF‐CBT interventions as a whole, EMDR would rank third, with only TF‐CBT and play therapy above it. Although EMDR had lower direct intervention costs, the effectiveness data used in the model meant that patients receiving TF‐CBT and play therapy were more likely to achieve remission than those receiving EMDR, relative to no treatment. For EMDR, this resulted in higher long‐term health state costs and lower QALYs, making it less cost‐effective. The deterministic sensitivity analysis showed that when using alternative values for the risk of relapse, the results remained robust. In the probabilistic sensitivity analysis, cognitive therapy remained the most cost‐effective intervention in all scenarios.

## 4 Discussion

This review of the clinical and cost‐effectiveness of EMDR for treatment and prevention of PTSD in children and adolescents identified eight relevant trials and one cost‐effectiveness study. Previous NICE guidance (NICE 2018) included only one of these eight studies (de Roos et al. 2017), highlighting the sizable amount of new evidence identified and analysed by the current review. No other systematic review has been published to date on the clinical and cost‐effectiveness of EMDR within this specific population of children and adolescents with PTSD or subclinical PTSD.

All of the studies investigating clinical effectiveness were at high or moderate risk of bias. Most compared EMDR with waitlist/usual care, with only two comparing EMDR with TF‐CBT. Meta‐analysis of data from five studies was feasible for the comparison of EMDR versus waitlist/usual care/no treatment and found that, for children and adolescents who received treatment more than 3 months after a traumatic event, EMDR was significantly more effective than waitlist/usual care/no treatment in reducing PTSD symptoms, regardless of the trauma (type and frequency) and age of the participants, or the details of the EMDR approach. This was measured at the end of treatment using self‐report scales, with a SMD of 1.57 (95% CrI 0.07–3.21).

Evidence from two studies comparing EMDR with TF‐CBT demonstrated that both therapies improved symptoms for children and adolescents with PTSD, but there was no significant difference between the two groups at follow‐up. One study was assessed to have a high risk of bias (Jaberghaderi et al. 2019) and one to have a moderate risk of bias (de Roos et al. 2017).

In terms of other outcomes, such as rates of discontinuations, adverse events and impact on anxiety and depression, the trend was also for there to be no significant difference between EMDR and TF‐CBT. There was no significant difference between EMDR and waitlist/usual care/no treatment for discontinuations and adverse events, but there was superiority of EMDR in terms of anxiety and depression. Further research is needed to better understand the factors that influence treatment discontinuation in children and adolescents receiving EMDR. Our review also identified two studies regarding the prevention of PTSD. Analysis of EMDR for this group (compared with usual care) is new: No studies were identified at the time of the previous NICE review or have been published in any other review.

The clinical effectiveness review therefore identified a number of RCTs demonstrating the consistent efficacy of EMDR for PTSD in a range of child and adolescent populations, using several different approaches to EMDR delivery, and in a number of different settings, using a number of different outcome measures. However, the evidence base is still relatively limited: It is at a high or moderate risk of bias, uses only self‐report outcome measures and is very limited in terms of active comparisons (only two trials comparing EMDR with the most common therapy of choice for child or adolescent PTSD, TF‐CBT) and prevention (as opposed to treatment). The unblinded design of studies introduces potential bias. However, while blinding of most key persons in RCTs of psychological interventions may not be feasible (Juul et al. 2021), outcome assessors might be blinded, but only when using clinician‐assessed tools.

A notable gap in the literature was the absence of within‐trial cost‐effectiveness analyses. This limitation hindered a comprehensive understanding of the cost–benefit profile of these interventions. The cost‐effectiveness review was based on a single modelling‐based cost‐effectiveness analysis which was conducted using a hybrid decision‐analytic model. The analysis found CBT to be the most cost‐effective intervention. However, it is important to note that the model's assumptions and the limited availability of cost data may influence the results. In terms of limitations of the review processes, the search applied a date limit of 2018 and used a previous review as the source for any relevant trials published before that date. Best practice would have been to conduct a full search without date limits. However, both the current review and the original NICE review applied exactly the same criteria, identified some overlap of evidence, and the current review also applied standard supplementary search techniques, such as reference checking, in order to identify any earlier trials that both reviews might have missed. It should be noted that all included trials were published before 2022 and therefore conducted in populations meeting the DSM and ICD criteria at the point in time of the study. An update search was conducted in January 2025 on the EMDR research publications database, which catalogues all peer‐reviewed literature relating to EMDR, and no new RCTs in children and adolescents with trauma were identified.

Further research is needed in several areas. High‐quality and better‐reported RCTs would be beneficial, including adequate blinding wherever possible, and evidence regarding longer term follow‐up is currently absent from the literature. Studies investigating the effectiveness of EMDR for prevention and early intervention in children and adolescents are required. Future research should also prioritise conducting within‐trial cost‐effectiveness analyses to provide more robust evidence on the economic value of different interventions for PTSD in young people. Additionally, further research is needed to refine model assumptions and incorporate more precise cost data to enhance the reliability of the findings. Despite these limitations, this review suggests potential benefits of EMDR for treating PTSD in children and adolescents.

## 5 Conclusion

EMDR was demonstrated to be effective in reducing PTSD symptoms in children and adolescents, particularly when compared with waitlist/usual care. However, the evidence base is limited, and more high‐quality RCTs are needed to confirm these findings and explore their broader applications. In addition, future research should prioritise within‐trial cost‐effectiveness analyses to provide a more comprehensive understanding of the cost–benefit profile of EMDR.

## Author Contributions

All authors contributed to the conceptualisation of the systematic review and contributed to the writing of the manuscript, including reviewing draft versions. Anthea Sutton (A.S.) led the systematic review, developed and performed the literature searches, screened studies, extracted data and drafted and finalised the manuscript. Christopher Carroll (C.C.) screened the studies, extracted data, conducted risk of bias assessments, synthesised data and drafted and finalised the manuscript. Emma Simpson (E.S.) screened the studies, extracted data, conducted risk of bias assessments and synthesised data. Jessica Forsyth (J.F.) conducted the statistical analysis. Annabel Rayner (A.R.) conducted the cost‐effectiveness analysis. Shijie Ren (S.R.) provided methodological advice. Matthew Franklin (M.F.) provided methodological advice. Emily Wood (E.W.) provided EMDR expertise and resolved screening queries and discrepancies.

## Funding

This study was supported by EMDR UK Association.

## Conflicts of Interest

This study is funded by the EMDR UK Association. Since October 2024, Anthea Sutton has a role as Research and Academic Liaison for EMDR UK but did not do so at the time of conducting this review.

## Supporting information


**Appendix S1:** PRISMA 2020 Checklist.
**Appendix S2:** Search strategies.
**Appendix S3:** Methods of data synthesis for clinical effectiveness.
**Appendix S4:** Other outcomes data.

## Data Availability

All data extracted is included in the manuscript and Supporting Information.
