# Supplementary material for: Clinical and Cost‐Effectiveness of Eye Movement Desensitisation and Reprocessing for Post‐Traumatic Stress Disorder in Children and Adolescents: A Systematic Review and Meta‐Analysis
Source: Clin Psychol Psychother. 2025 Dec 4;32(6):e70186. doi: 10.1002/cpp.70186 (PMC12676259; doi:10.1002/cpp.70186)
Supplement: Supplementary file 1 — Appendix S1: PRISMA 2020 Checklist. Appendix S2: Search strategies. Appendix S3: Methods of data synthesis for clinical effectiveness. Appendix S4: Other outcomes data. [file CPP-32-e70186-s001.docx]

**Supplementary file**

Appendix 1: PRISMA 2020 Checklist

| **Section and Topic** | **Item #** | **Checklist item** | **Location where item is reported** |
| --- | --- | --- | --- |
| **TITLE** | | |  |
| Title | 1 | Identify the report as a systematic review. | Title page, 1 |
| **ABSTRACT** | | |  |
| Abstract | 2 | See the PRISMA 2020 for Abstracts checklist. | Title page, 1 |
| **INTRODUCTION** | | |  |
| Rationale | 3 | Describe the rationale for the review in the context of existing knowledge. | Page 2 |
| Objectives | 4 | Provide an explicit statement of the objective(s) or question(s) the review addresses. | Page 2 |
| **METHODS** | | |  |
| Eligibility criteria | 5 | Specify the inclusion and exclusion criteria for the review and how studies were grouped for the syntheses. | Pages 3-4, Table 1 |
| Information sources | 6 | Specify all databases, registers, websites, organisations, reference lists and other sources searched or consulted to identify studies. Specify the date when each source was last searched or consulted. | Pages 3 |
| Search strategy | 7 | Present the full search strategies for all databases, registers and websites, including any filters and limits used. | Appendix 2 |
| Selection process | 8 | Specify the methods used to decide whether a study met the inclusion criteria of the review, including how many reviewers screened each record and each report retrieved, whether they worked independently, and if applicable, details of automation tools used in the process. | Page 4 |
| Data collection process | 9 | Specify the methods used to collect data from reports, including how many reviewers collected data from each report, whether they worked independently, any processes for obtaining or confirming data from study investigators, and if applicable, details of automation tools used in the process. | Page 4 |
| Data items | 10a | List and define all outcomes for which data were sought. Specify whether all results that were compatible with each outcome domain in each study were sought (e.g. for all measures, time points, analyses), and if not, the methods used to decide which results to collect. | Page 4-5 |
|  | 10b | List and define all other variables for which data were sought (e.g. participant and intervention characteristics, funding sources). Describe any assumptions made about any missing or unclear information. | Page 4-5 |
| Study risk of bias assessment | 11 | Specify the methods used to assess risk of bias in the included studies, including details of the tool(s) used, how many reviewers assessed each study and whether they worked independently, and if applicable, details of automation tools used in the process. | Page 4-5 |
| Effect measures | 12 | Specify for each outcome the effect measure(s) (e.g. risk ratio, mean difference) used in the synthesis or presentation of results. | Page 5 and Appendix 3 |
| Synthesis methods | 13a | Describe the processes used to decide which studies were eligible for each synthesis (e.g. tabulating the study intervention characteristics and comparing against the planned groups for each synthesis (item #5)). | Page 5 |
|  | 13b | Describe any methods required to prepare the data for presentation or synthesis, such as handling of missing summary statistics, or data conversions. | Page 5 and Appendix 3 |
|  | 13c | Describe any methods used to tabulate or visually display results of individual studies and syntheses. | Page 5 and Appendix 3 |
|  | 13d | Describe any methods used to synthesize results and provide a rationale for the choice(s). If meta-analysis was performed, describe the model(s), method(s) to identify the presence and extent of statistical heterogeneity, and software package(s) used. | Page 5 and Appendix 3 |
|  | 13e | Describe any methods used to explore possible causes of heterogeneity among study results (e.g. subgroup analysis, meta-regression). | Page 5 and Appendix 3 |
|  | 13f | Describe any sensitivity analyses conducted to assess robustness of the synthesized results. | Page 5 and Appendix 3 |
| Reporting bias assessment | 14 | Describe any methods used to assess risk of bias due to missing results in a synthesis (arising from reporting biases). | Page 5 and Appendix 3 |
| Certainty assessment | 15 | Describe any methods used to assess certainty (or confidence) in the body of evidence for an outcome. | Not reported |
| **RESULTS** | | |  |
| Study selection | 16a | Describe the results of the search and selection process, from the number of records identified in the search to the number of studies included in the review, ideally using a flow diagram. | Pages 5-6 and Figure 1 |
|  | 16b | Cite studies that might appear to meet the inclusion criteria, but which were excluded, and explain why they were excluded. | Appendix 4 |
| Study characteristics | 17 | Cite each included study and present its characteristics. | Pages 7-8 and Tables 2 and 3 |
| Risk of bias in studies | 18 | Present assessments of risk of bias for each included study. | Page 8 and Figure 2 |
| Results of individual studies | 19 | For all outcomes, present, for each study: (a) summary statistics for each group (where appropriate) and (b) an effect estimate and its precision (e.g. confidence/credible interval), ideally using structured tables or plots. | Pages 9-13, Table 4, Figure 3, and Appendix 5 |
| Results of syntheses | 20a | For each synthesis, briefly summarise the characteristics and risk of bias among contributing studies. | Figure 3 |
|  | 20b | Present results of all statistical syntheses conducted. If meta-analysis was done, present for each the summary estimate and its precision (e.g. confidence/credible interval) and measures of statistical heterogeneity. If comparing groups, describe the direction of the effect. | Pages 9-13, Table 4 and Figure 3 |
|  | 20c | Present results of all investigations of possible causes of heterogeneity among study results. | Pages 9-12, and Discussion |
|  | 20d | Present results of all sensitivity analyses conducted to assess the robustness of the synthesized results. | N/A |
| Reporting biases | 21 | Present assessments of risk of bias due to missing results (arising from reporting biases) for each synthesis assessed. | Not reported |
| Certainty of evidence | 22 | Present assessments of certainty (or confidence) in the body of evidence for each outcome assessed. | Not reported |
| **DISCUSSION** | | |  |
| Discussion | 23a | Provide a general interpretation of the results in the context of other evidence. | Pages 13-14 |
|  | 23b | Discuss any limitations of the evidence included in the review. | Page 14 |
|  | 23c | Discuss any limitations of the review processes used. | Pages 14 |
|  | 23d | Discuss implications of the results for practice, policy, and future research. | Page 14-15 |
| **OTHER INFORMATION** | | |  |
| Registration and protocol | 24a | Provide registration information for the review, including register name and registration number, or state that the review was not registered. | Protocol registered, page 2 and Title page |
|  | 24b | Indicate where the review protocol can be accessed, or state that a protocol was not prepared. | CRD: 42023463360 |
|  | 24c | Describe and explain any amendments to information provided at registration or in the protocol. | None |
| Support | 25 | Describe sources of financial or non-financial support for the review, and the role of the funders or sponsors in the review. | Title page |
| Competing interests | 26 | Declare any competing interests of review authors. | Within journal |
| Availability of data, code and other materials | 27 | Report which of the following are publicly available and where they can be found: template data collection forms; data extracted from included studies; data used for all analyses; analytic code; any other materials used in the review. | Tables and data all provided within output |

**Appendix 2: Search Strategies**

**MEDLINE**

Ovid MEDLINE(R) and Epub Ahead of Print, In-Process, In-Data-Review & Other Non-Indexed Citations and Daily <1946 to September 14, 2023>

1 exp Stress Disorders, Post-Traumatic/ 41583

2 PTSD.ti,ab. 32537

3 moral* injur*.ti,ab. 676

4 exp Combat Disorders/ 3219

5 ((combat or battle or conflict or war or wars) adj5 (stress or disorder* or neuros*)).ti,ab. 5242

6 war syndrome*.ti,ab. 309

7 (shell shock* or shellshock* or shell-shock*).ti,ab. 156

8 exp Psychological Trauma/ 1982

9 exp Stress Disorders, Traumatic/ 46034

10 exp Stress Disorders, Traumatic, Acute/ 541

11 ((traumatic or acute) adj stress disorder*).ti,ab. 16710

12 (railway spine or (rape adj2 trauma*) or reexperienc* or re experienc* or torture syndrome or traumatic neuros* or traumatic stress).ti,ab. 23156

13 (trauma* and (avoidance or grief or horror or death* or nightmare* or night mare* or emotion*)).ti,ab. 47697

14 (posttraumatic* or post traumatic* or post-traumatic* or stress disorder* or acute stress or asd or desnos or combat syndrome or concentration camp syndrome or extreme stress or flashback* or flash back* or hypervigilan* or hypervigilen* or psych* stress or psych* trauma* or psycho?trauma* or psychotrauma* or posttrauma* or traumagenic* or traumatic stress*).ti,ab. 143852

15 (sexual adj2 trauma).ti,ab. 1489

16 exp Sexual Trauma/ 133

17 or/1-16 199781

18 exp Eye Movement Desensitization Reprocessing/ 382

19 (EMDR or eye movement desensiti?ation reprocessing).tw. 799

20 "eye movement desensiti?ation and processing".tw. 4

21 18 or 19 or 20 871

22 17 and 21 685

23 (2018* or 2019* or 2020* or 2021* or 2022* or 2023*).dt. 8267307

24 22 and 23 324

**Embase**

Embase <1974 to 2023 Week 36>

1 exp posttraumatic stress disorder/ 80336

2 PTSD.ti,ab. 42375

3 moral* injur*.ti,ab. 732

4 exp combat stress/ 86

5 ((combat or battle or conflict or war or wars) adj5 (stress or disorder* or neuros*)).ti,ab. 6214

6 war syndrome*.ti,ab. 367

7 (shell shock* or shellshock* or shell-shock*).ti,ab. 154

8 exp psychotrauma/ 11517

9 exp acute stress disorder/ 1782

10 ((traumatic or acute) adj stress disorder*).ti,ab. 21855

11 (railway spine or (rape adj2 trauma*) or reexperienc* or re experienc* or torture syndrome or traumatic neuros* or traumatic stress).ti,ab. 30286

12 (trauma* and (avoidance or grief or horror or death* or nightmare* or night mare* or emotion*)).ti,ab. 67617

13 (posttraumatic* or post traumatic* or post-traumatic* or stress disorder* or acute stress or asd or desnos or combat syndrome or concentration camp syndrome or extreme stress or flashback* or flash back* or hypervigilan* or hypervigilen* or psych* stress or psych* trauma* or psycho?trauma* or psychotrauma* or posttrauma* or traumagenic* or traumatic stress*).ti,ab. 184551

14 (sexual adj2 trauma).ti,ab. 1996

15 exp sexual trauma/ 387

16 exp acute stress/ 7018

17 exp behavioral stress/ 1066

18 exp emotional stress/ 27866

19 exp critical incident stress/ 116

20 exp mental stress/ 201010

21 or/1-20 448749

22 exp "eye movement desensitization and reprocessing"/ 697

23 (EMDR or eye movement desensiti?ation reprocessing).tw. 1055

24 "eye movement desensiti?ation and processing".tw. 3

25 22 or 23 or 24 1331

26 21 and 25 1025

27 (2018* or 2019* or 2020* or 2021* or 2022* or 2023*).dc. 10765299

28 26 and 27 501

**PsycINFO**

APA PsycInfo <1806 to September Week 1 2023>

1 exp Posttraumatic Stress Disorder/ 40469

2 PTSD.ti,ab. 39343

3 moral* injur*.ti,ab. 720

4 ((combat or battle or conflict or war or wars) adj5 (stress or disorder* or neuros*)).ti,ab. 6166

5 war syndrome*.ti,ab. 90

6 (shell shock* or shellshock* or shell-shock*).ti,ab. 236

7 exp Acute Stress Disorder/ 681

8 exp Combat Experience/ 3319

9 exp Emotional Trauma/ 16356

10 exp Posttraumatic Stress/ 2124

11 exp Traumatic Neurosis/ 310

12 exp Trauma/ 81027

13 exp Psychological Stress/ 9773

14 exp Chronic Stress/ 3348

15 ((traumatic or acute) adj stress disorder*).ti,ab. 14129

16 (railway spine or (rape adj2 trauma*) or reexperienc* or re experienc* or torture syndrome or traumatic neuros* or traumatic stress).ti,ab. 22644

17 (trauma* and (avoidance or grief or horror or death* or nightmare* or night mare* or emotion*)).ti,ab. 32437

18 (posttraumatic* or post traumatic* or post-traumatic* or stress disorder* or acute stress or asd or desnos or combat syndrome or concentration camp syndrome or extreme stress or flashback* or flash back* or hypervigilan* or hypervigilen* or psych* stress or psych* trauma* or psycho?trauma* or psychotrauma* or posttrauma* or traumagenic* or traumatic stress*).ti,ab. 100318

19 (sexual adj2 trauma).ti,ab. 2310

20 exp Sexual Abuse/ 23241

21 or/1-20 210008

22 exp Eye Movement Desensitization Therapy/ 1917

23 (EMDR or eye movement desensiti?ation reprocessing).tw. 2239

24 "eye movement desensiti?ation and processing".tw. 4

25 22 or 23 or 24 2354

26 21 and 25 1680

27 limit 26 to yr="2018 -Current" 538

**Cochrane**

Search Name:

Date Run: 15/09/2023 15:05:55

Comment:

ID Search Hits

#1 MeSH descriptor: [Stress Disorders, Post-Traumatic] explode all trees 3689

#2 (PTSD):ti,ab,kw (Word variations have been searched) 5727

#3 moral* injur*:ti,ab,kw 204

#4 MeSH descriptor: [Combat Disorders] explode all trees 143

#5 ((combat or battle or conflict or war or wars) near/5 (stress or disorder* or neuros*)):ti,ab,kw 544

#6 war syndrome*:ti,ab,kw 165

#7 (shell shock* or shellshock* or shell-shock*):ti,ab,kw 9

#8 MeSH descriptor: [Psychological Trauma] explode all trees 134

#9 MeSH descriptor: [Stress Disorders, Traumatic] explode all trees 3882

#10 MeSH descriptor: [Stress Disorders, Traumatic, Acute] explode all trees 58

#11 MeSH descriptor: [Stress, Psychological] explode all trees 7999

#12 ((traumatic or acute) NEXT stress disorder*):ti,ab,kw 3050

#13 ("railway spine" or (rape near/2 trauma*) or reexperienc* or re experien* or "torture

syndrome" or traumatic neuros* or "traumatic stress"):ti,ab,kw 7327

#14 (trauma* and (avoidance or grief or horror or death* or nightmare* or night mare* or

emotion*)):ti,ab,kw 4758

#15 (posttraumatic* or post traumatic* or stress disorder* or "acute stress" or asd or

desnos or ("combat syndrome" or "concentration camp syndrome" or

"extreme stress" or flashback* or flash back* or hypervigilan* or hypervigilen* or psych*

stress or psych* trauma* or psychotrauma* or psychotrauma*) or (posttrauma* or

traumagenic* or traumatic stress*)):ti,ab,kw 43872

#16 (sexual NEAR/2 trauma):ti,ab,kw 119

#17 MeSH descriptor: [Sexual Trauma] explode all trees 13

#18 {OR #1-#17} 50803

#19 MeSH descriptor: [Eye Movement Desensitization Reprocessing] explode all trees 100

#20 (EMDR or eye movement desensiti?ation reprocessing):ti,ab,kw 579

#21 ("eye movement desensitization and processing" or "eye movement desensitization and processing"):ti,ab,kw 1

#22 #19 or #20 or #21 579

#23 #18 and #22 with Cochrane Library publication date Between Jan 2018 and Sep 2023 291

**CINAHL**

Accessibility Information and TipsPrint Search History

Friday, September 15, 2023 4:38:53 PM

# Query Limiters/Expanders Last Run Via Results

S22 S20 AND S21 Expanders - Apply equivalent subjects

Search modes - Boolean/Phrase Interface - EBSCOhost Research Databases

Search Screen - Advanced Search

Database - CINAHL 169

S21 Limiters - Published Date: 20180101-20231231

Expanders - Apply equivalent subjects

Search modes - Boolean/Phrase Interface - EBSCOhost Research Databases

Search Screen - Advanced Search

Database - CINAHL 2,564,970

S20 S15 AND S19 Expanders - Apply equivalent subjects

Search modes - Boolean/Phrase Interface - EBSCOhost Research Databases

Search Screen - Advanced Search

Database - CINAHL 404

S19 S16 OR S17 OR S18 Expanders - Apply equivalent subjects

Search modes - Boolean/Phrase Interface - EBSCOhost Research Databases

Search Screen - Advanced Search

Database - CINAHL 594

S18 "eye movement desensiti?ation and processing" Expanders - Apply equivalent subjects

Search modes - Boolean/Phrase Interface - EBSCOhost Research Databases

Search Screen - Advanced Search

Database - CINAHL 1

S17 (EMDR or eye movement desensiti?ation reprocessing) Expanders - Apply equivalent subjects

Search modes - Boolean/Phrase Interface - EBSCOhost Research Databases

Search Screen - Advanced Search

Database - CINAHL 485

S16 (MH "Eye Movement Desensitization and Reprogramming") Expanders - Apply equivalent subjects

Search modes - Boolean/Phrase Interface - EBSCOhost Research Databases

Search Screen - Advanced Search

Database - CINAHL 385

S15 S1 OR S2 OR S3 OR S4 OR S5 OR S6 OR S7 OR S8 OR S9 OR S10 OR S11 OR S12 OR S13 OR S14 Expanders - Apply equivalent subjects

Search modes - Boolean/Phrase Interface - EBSCOhost Research Databases

Search Screen - Advanced Search

Database - CINAHL 181,127

S14 (MH "Sexual Trauma+") OR (MH "Military Sexual Trauma") Expanders - Apply equivalent subjects

Search modes - Boolean/Phrase Interface - EBSCOhost Research Databases

Search Screen - Advanced Search

Database - CINAHL 119

S13 TI (sexual N2 trauma) OR AB (sexual N2 trauma) Expanders - Apply equivalent subjects

Search modes - Boolean/Phrase Interface - EBSCOhost Research Databases

Search Screen - Advanced Search

Database - CINAHL 1,136

S12 TI ( (posttraumatic* or post traumatic* or post-traumatic* or stress disorder* or acute stress or asd or desnos or combat syndrome or concentration camp syndrome or extreme stress or flashback* or flash back* or hypervigilan* or hypervigilen* or psych* stress or psych* trauma* or psycho?trauma* or psychotrauma* or posttrauma* or traumagenic* or traumatic stress*) ) OR AB ( (posttraumatic* or post traumatic* or post-traumatic* or stress disorder* or acute stress or asd or desnos or combat syndrome or concentration camp syndrome or extreme stress or flashback* or flash back* or hypervigilan* or hypervigilen* or psych* stress or psych* trauma* or psycho?trauma* or psychotrauma* or posttrauma* or traumagenic* or traumatic stress*) ) Expanders - Apply equivalent subjects

Search modes - Boolean/Phrase Interface - EBSCOhost Research Databases

Search Screen - Advanced Search

Database - CINAHL 63,498

S11 TI ( (trauma* and (avoidance or grief or horror or death* or nightmare* or night mare* or emotion*)) ) OR AB ( (trauma* and (avoidance or grief or horror or death* or nightmare* or night mare* or emotion*)) ) Expanders - Apply equivalent subjects

Search modes - Boolean/Phrase Interface - EBSCOhost Research Databases

Search Screen - Advanced Search

Database - CINAHL 17,332

S10 TI ( (railway spine or (rape N2 trauma*) or reexperienc* or re experienc* or torture syndrome or traumatic neuros* or traumatic stress) ) OR AB ( (railway spine or (rape N2 trauma*) or reexperienc* or re experienc* or torture syndrome or traumatic neuros* or traumatic stress) ) Expanders - Apply equivalent subjects

Search modes - Boolean/Phrase Interface - EBSCOhost Research Databases

Search Screen - Advanced Search

Database - CINAHL 12,281

S9 TI ( ((traumatic or acute) N1 stress disorder*) ) OR AB ( ((traumatic or acute) N1 stress disorder*) ) Expanders - Apply equivalent subjects

Search modes - Boolean/Phrase Interface - EBSCOhost Research Databases

Search Screen - Advanced Search

Database - CINAHL 7,493

S8 (MH "Stress, Psychological+") Expanders - Apply equivalent subjects

Search modes - Boolean/Phrase Interface - EBSCOhost Research Databases

Search Screen - Advanced Search

Database - CINAHL 103,646

S7 (MH "Psychological Trauma+") Expanders - Apply equivalent subjects

Search modes - Boolean/Phrase Interface - EBSCOhost Research Databases

Search Screen - Advanced Search

Database - CINAHL 2,737

S6 TI ( (shell shock* or shellshock* or shell-shock*) ) OR AB ( (shell shock* or shellshock* or shell-shock*) ) Expanders - Apply equivalent subjects

Search modes - Boolean/Phrase Interface - EBSCOhost Research Databases

Search Screen - Advanced Search

Database - CINAHL 61

S5 TI war syndrome* OR AB war syndrome* Expanders - Apply equivalent subjects

Search modes - Boolean/Phrase Interface - EBSCOhost Research Databases

Search Screen - Advanced Search

Database - CINAHL 127

S4 TI ( ((combat or battle or conflict or war or wars) N5 (stress or disorder* or neuros*)) ) OR AB ( ((combat or battle or conflict or war or wars) N5 (stress or disorder* or neuros*)) ) Expanders - Apply equivalent subjects

Search modes - Boolean/Phrase Interface - EBSCOhost Research Databases

Search Screen - Advanced Search

Database - CINAHL 2,279

S3 TI moral* injur* OR AB moral* injur* Expanders - Apply equivalent subjects

Search modes - Boolean/Phrase Interface - EBSCOhost Research Databases

Search Screen - Advanced Search

Database - CINAHL 454

S2 TI PTSD OR AB PTSD Expanders - Apply equivalent subjects

Search modes - Boolean/Phrase Interface - EBSCOhost Research Databases

Search Screen - Advanced Search

Database - CINAHL 14,419

S1 (MH "Stress Disorders, Post-Traumatic+") Expanders - Apply equivalent subjects

Search modes - Boolean/Phrase Interface - EBSCOhost Research Databases

Search Screen - Advanced Search

Database - CINAHL 27,213

**PTSDpubs**

MAINSUBJECT.EXACT.EXPLODE("EMDR") OR title(EMDR OR eye movement desensiti?ation reprocessing OR "eye movement desensiti?ation and processing") OR abstract(EMDR OR eye movement desensiti?ation reprocessing OR "eye movement desensiti?ation and processing")Limits applied (2018-2023)

Hand searching of key journals and websites Journal of EMDR Practice and Research, Current Approaches in Psychiatry, American Journal of Applied Psychology, Clinical Neuropsychiatry, Scientific Reports, European Journ- al of Therapeutics, Journal of Korean Neuropsychiatric Association, Psychology and Behavioral Science, Open Journal of Social Sciences, IberoAmerican Journal of Psychotrauma and Dissociation. Relevant organisation websites were searched: National Institute of Health and Care Excellence (NICE); American Psychological Association; EMDR Europe; EMDR Association of Australia; EMDR Institute of Israel.

# **Appendix 3: Methods of data synthesis for clinical effectiveness**

In order for meta-analyses to be conducted, there needed to be at least three studies providing data for a comparison. Studies could be from our review, or the review on which NICE guidance was based [(National Institute for and Care, 2018)]. In order to be included in a meta-analysis, a study had to include both mean and SD for the change in PTSD from pre- to post-treatment, or these data had to be calculable.

Comparative effectiveness was evaluated using pairwise meta-analysis (MA).**(Dias et al., 2013)** Five comparisons were conducted, comparing direct evidence of treatment effect from multiple studies. The analyses were conducted using a Bayesian Markov Chain Monte Carlo (MCMC) approach using a random effects model to account for heterogeneity in treatment effects across studies.**(Dias et al., 2013)**

The outcome considered was the change in PTSD symptoms before and after treatment.

For the purposes of this review, positive change in mean indicated improvement, and negative change in mean indicated worsening of symptoms.

There were no possible meta-analyses for prevention, or for early treatment. All meta-analyses were for adults with PTSD given delayed treatment (i.e. three months or more following trauma). Pairwise meta-analyses were conducted for the following comparisons:

EMDR vs. TF-CBT, PTSD self-report, follow-up post-treatment;

EMDR vs. TF-CBT, PTSD clinician-rated, follow-up post-treatment;

EMDR vs. wait-list/usual care, PTSD self-report, follow-up post-treatment;

EMDR vs. wait-list/usual care, PTSD self-report, follow-up three months following treatment;

The scoring of PTSD symptoms was conducted using different scoring methods, studies reporting any validated PTSD scale were included. To enable the use of studies using different scoring methods within a single pairwise MA, standardised mean differences (SMDs) were calculated for each study. The use of SMDs is based on the assumption that all scoring scales are quantifying the same treatment effect and can be transformed onto a common scale by dividing the mean difference between the intervention and comparator within each study by the standard deviation of the difference. Raw data extracted from study results in the form of means/standard deviation/confidence intervals were used to evaluate the SMD and subsequently the standard error (SE) for each study using Hedge’s correction.**(Higgins and Altman, 2008)** Using intervention 1 as the reference, the SMD for the interventions in arm $t=2$, at follow-up $f=1,2$ is given by

$${SMD}_{t,f}=c\cdot\frac{\mu_{f, t}-\mu_{f, 1}}{S}$$

$$S=\sqrt{\frac{{(n}_{1}-1)S_{1}^{2}+{(n}_{t}-1)S_{t}^{2}}{n_{1}+n_{t}-2}}$$

$$c=1-\frac{3}{4\left( n_{1}+n_{t} \right)-9}$$

where $\mu_{f, t}$ is the change in score before and after treatment for arm t at follow-up f; $S$ is the within group standard deviation pooled across groups and$c$ is Hedges’ correction factor. The standard error of the SMD is given by

$$SE({SMD}_{t,f})=\sqrt{c^{2}\left( \frac{n_{1}+n_{t}}{n_{1}n_{t}}+\frac{{{SMD}_{t,f}}^{2}}{2(n_{1}+n_{t})} \right)}$$

Where the 95% confidence intervals were provided as opposed to the standard deviation, the standard deviation was evaluated using,

$$SD=\sqrt{n}\frac{CI_{upper}-CI_{lower}}{C},$$

where $n$ is the number of particpants in the study arm, $CI_{upper}$ and $CI_{lower}$ are the upper and lower 95% confidence intervals respectively and $C$ was chosen to be 3.92 for all studies with n>60.**(Higgins and Altman, 2008)** For studies which only reported pre- and post-treatment scores, the change in PTSD symptoms score before and after treatment was calculated based on the reported pre- and post-treatment scores and standard error was calculated assuming the correlation is 0.5.

## ***Statistical model for the meta-analysis***

Let $y_{ik}$ denote the SMD of arm $k$ of trial $i$ where $k=1,\ldots,na$ and $i=1,\ldots,ns$, with variance $V_{ik}$. We assume that the treatment effects are normally distributed such that,

$$y_{ik}\mathcal{\sim N}\left( \theta_{ik}, V_{ik} \right),$$

where $\boldsymbol{\theta}$ are the parameters of interest. The individual $\theta_{ik}$ are modelled using the identity link function as they are continuous and over the entire real line,

$$\theta_{ik}=\delta_{i,1k}.$$

To allow for heterogeneity of treatment effects across studies, a random effects model was assumed. The random effects model is structured such that all individual study treatment effects, $\delta_{i,1k}$ arise from a common normal distribution centred about a mean treatment effect with some variance, $\tau^{2}$,

$$\delta_{i,1k}\mathcal{\sim N(}d_{t_{i1}t_{ik}}, \tau^{2})$$

where $d_{t_{i1}t_{ik}}$ represents the mean effect of treatment $k$ of study $i$ ($t_{ik}$) compared to the treatment in arm 1 of study $i$($t_{i1}$).

As only pairwise meta-analyses were conducted, $k=1,2$, the subscripts within the model can be further simplified to the following,

$$y_{i2}\mathcal{\sim N}\left( \theta_{i2}, V_{i2} \right),$$

$$\theta_{i2}=\delta_{i,12},$$

$$\delta_{i,12}\mathcal{\sim N}\left( d_{t_{i1}t_{i2}}, \tau^{2} \right),$$

where $y_{i2}$ is the SMD between EMDR (subscript 2) and the comparator treatment (subscript 1) and $\delta_{i,12}$ is the study-specific treatment SMD between EMDR and the comparator.

Parameters were estimated using a Bayesian framework, as such non-informative priors were chosen for the between-study standard deviation of treatment effects, $\tau$, and the mean treatment effect, $d_{t_{i1}, t_{i2}}$:

- $\tau\sim\mathcal{U}\left( 0,5 \right)\cdot\frac{\sqrt{3}}{\pi}$
- $d_{t_{i1}, t_{i2}}\mathcal{\sim N(}{0,100}^{2})$

In cases where there was not sufficient evidence to inform inference on $\tau$, a more informative prior was chosen for the between study standard deviation of treatment effects,

$$\tau\sim\mathrm{lognormal}\left( 0.13, 0.249 \right).$$

This prior was derived using information from relevant comparisons with higher numbers of studies.

All analyses were conducted using the freely available software WinBUGS **(Lunn et al., 2000)**via the R package, R2WinBUGS **(Sturtz et al., 2005).** Convergence to the target posterior was assessed using the Gelman-Rubin statistic.**(Brooks and Gelman, 1998)** All simulations appeared to converge within 20,000 iterations and so a burn-in period of 20,000 samples was chosen for all analyses. A further 10,000 iterations of the Markov chain were retained to estimate parameters of interest after thinning the chain samples by a factor of 10.

Results are presented alongside the posterior median treatment effects and 95% credible intervals (CrI). Effect sizes were graded using Cohen’s categories; not substantial (SMD<0.2), small (0.2$\leq$SMD<0.5), medium (0.5$\leq$SMD<0.8), large (0.8$\leq$SMD).**(Cohen, 2013)** Study heterogeneity was graded and interpreted according to the categories introduced in Ren *et al.* 2018.(Ren et al., 2018)

**Appendix 4: Other outcomes data**

Table S1 Discontinuations

| **Author, year**  **(study name)** | **Interventions** | **Treatment duration (as reported)** | **Treatment Discontinuations, n/N (%)** | **Study discontinuations** |
| --- | --- | --- | --- | --- |
| **Children, treatment, TF-CBT, delayed** | | |  |  |
| Jaberghaderi, 2019 | EMDR | 4-12 weeks (between 3 and 12 sessions of 45 to 60 minutes) | 11/40 (27.5) | 16/40 |
|  | TF-CBT | 4-12 weeks (up to 12 sessions) | 11/40 (27.5) | 15/40 |
| **Children, treatment, wait list/usual care, delayed** | | | |  |
| Banoglu, 2021 | EMDR-GP/C | Unclear (each participant joined 3-4 group sessions till (s)hereported a SUD rating of 0 and e every group session was held with 8  participants on average. An EMDR-GP/C session took 90-120 minutes) | 0/42 | 5/47 |
|  | WL | NA | NA | 28/47 |
| Jaberghaderi, 2019 | EMDR | 4-12 weeks (between 3 and 12 sessions of 45 to 60 minutes) | 11/40 (27.5) | 16/40 |
|  | WL | NA | NA | 6/59 |
| Jimenez, 2020 | EMDR-PRECI | Average of 4.68 60-minute individual sessions provided two or three times a week (depending on availability of participants) | 0/16 | 0 / 16 |
|  | TAU** | Average of 12.6 60-minute individual sessions provided once a week | 0/16 | 4/16 |
| Karadag, 2021 | EMDR booklet | 6 days (intervention group carried out the activities in the guide three times in total, once every 2 days) | NR/88 (-) | 36/88 |
|  | WL | NA | NA | 45/90 |
| Molero, 2019 | EMDR-IGTP-OTS | 3 days (9 treatment sessions [1st session average 95 minutes, other sessions average 48 minutes]; three times a day during three consecutive days) | 0/93 | 17/93 |
|  | No treatment | NA | NA | 16/91 |
| **Children, prevention, wait list/usual care, delayed** | | | |  |
| Meentken 2020, 2021 | EMDR | 3-4 weeks (weekly sessions; mean 3.5 (SD = 1.9) sessions) | 1/37 (2.7) | 9/37 |
|  | WL | NA | NA | 2/37 (2.7) |
| Osorio, 2018 | EMDR-IGTP-OTS | 2 days (6 treatment sessions [1st session average 106 minutes, other sessions average 53 minutes]; three times a day during two consecutive days) | 0/11 | 0/11 |
|  | No treatment | NA | NA | 0/12 |

EMDR: Eye Movement and Desensitization Reprocessing; GP/C: Group protocol with children; IGTP-OTS: Integrative Group Treatment Protocol-Ongoing Traumatic Stress; ImRs: Imagery Rescripting; NA: Not applicable; NR: Not reported; PRECI: Protocol for Recent Critical Incidents and Ongoing Traumatic Stress; PTSD: Post Traumatic Stress Disorder; TAU: Treatment as usual; TF-CBT: Trauma-focused CBT; WL: Wait list. ** TAU: psychological support, oriented to life plan and emotions management

Table S2 Adverse events

| **Author, year**  **(study name)** | **Intervention (n)** | **Follow-ups** | **Adverse events** |
| --- | --- | --- | --- |
| **Children, treatment, TF-CBT, delayed** | | | |
| Jaberghaderi, 2019 | EMDR (40) vs TF-CBT (40) | 2 weeks (post treatment) | Not reported |
| **Children, treatment, wait list/usual care, delayed** | | | |
| Banoglu, 2021 | EMDR-GP/C (47) vs WL (47) | Unclear | Not reported |
| Jaberghaderi, 2019 | EMDR (40) vs WL (59) | 2 weeks (post treatment) | Not reported |
| Jimenez, 2020 | EMDR-PRECI (16) vs TAU** (16) | 90 days | ‘No adverse effects or pregnancy complications were reported during EMDR  treatment or at a three-month post-treatment assessment’. |
| Karadag, 2021 | EMDR (booklet) (88) vs WL (90) | 4 weeks | Not reported |
| Molero, 2019 | EMDR-IGTP-OTS (93) vs no treatment (91) | 90 days | ‘No adverse effects were reported during treatment or at three-month  post-treatment assessment’. |
| **Children, prevention, wait list/usual care** | | | |
| Meentken 2020, 2021 | EMDR (37) vs WL (37) | 8 weeks | Not reported |
| Osorio, 2018 | EMDR-IGTP-OTS (12) vs no treatment (11) | 90 days | ‘No adverse effects were reported during treatment or at 90-day follow-up’. |

Table S3 Depression and anxiety

| **Author, year**  **(study name)** | **Interventions (n)** | **Follow-ups** | **Depression**  Mean change from baseline / pretherapy (estimated 95% CI) | **Anxiety** |
| --- | --- | --- | --- | --- |
| **Children, treatment, TF-CBT, delayed** | | | | |
| Jaberghaderi, 2019 | EMDR (40) vs TF-CBT (40) | 2 weeks (post treatment) | NR | NR |
| **Children, treatment, wait list/usual care, delayed** | | | | |
| Banoglu, 2021 | EMDR-GP/C (47) vs WL (47) | Unclear (post treatment) | Major Depression Inventory (MDI), Mean (SD), adjusted mean (SE)  EMDR: Baseline: 15.73, (11.01); Follow-up: 11.05 (SE=1.26)  WL: Baseline: 16.60 (11.22); Follow-up:  18.46 (SE=2.18)  When adjusted for the pre-treatment  scores, EMDR group had significantly lower depression score on average  (Mean = 11.05, SE = 1.26, 95% CI [8.40, 13.70]) than the control group  (Mean = 18.46, SE = 2.18, 95% CI [13.86, 23.05]). | NR |
| Jaberghaderi, 2019 | EMDR (40) vs WL (59) | 2 weeks (post treatment) | NR | NR |
| Jimenez, 2020 | EMDR-PRECI (16) vs TAU** (16) | 90 days | HADS, mean (SD), pre-treatment, 90 days:  EMDR-PRECI: 8.18 (4.32); 3.00 (2.65); mean change from baseline (SD) : 5.18 (4.07)  TAU: 5.58 (4.12); 4.00 (2.82); mean change from baseline (SD) : 1.58 (3.03)  Significant effects for time (F (1, 26) = 21.77, p <. 001, ηP2 =.456). Results also showed significant interaction effects between time and group. (F (1, 26) = 6.17,  p <.05, ηP2 =.192). | HADS, mean (SD), pre-treatment, 90 days:  EMDR-PRECI: 10.62 (4.48); 3.25 (2.64); mean change from baseline (SD) : 7.17 (4.19)  TAU: 9.08 (4.87); 7.91 (4.12); mean change from baseline (SD) : -1.17 (4.96)  Significant effect for time (F (1, 26) =  32.89, p <.001, ηP 2 =.559) and a significant interaction effect between time and group (F (1, 26) = 17.37, p <.001, ηP2=.401). |
| Karadag, 2021 | EMDR (booklet) (88) vs WL (90) | 4 weeks | NR | State-Trait Anxiety Inventory for Children (STAIC), mean (SD); baseline to 4 weeks:  EMDR:  State anxiety: 32.1 (9.1); 28.5 (8.3); mean (SD) change from baseline: 3.6 (9.55).  Trait Anxiety: 36.9 (7.9); 34.6 (6.7); mean (SD) change from baseline: 2.3 (8.06).  WL:  State Anxiety: 32.2 (7.7); 29 (89.9); mean (SD) change from baseline: 3.2 (9.81).  Trait Anxiety: 35.5 (8.07); 34.2 (9.8); mean (SD) change from baseline: 1.3 (9.89) |
| Molero, 2019 | EMDR-IGTP-OTS (93) vs no treatment (91) | 90 days | HADS, mean (SD), pre-treatment, 90 days:  EMDR-IGTP-OTS: 9.30 (3.06); 6.30 (4.37); mean change from baseline (SD) : 3.00 (4.21)  No treatment: 8.63 (4.18); 8.72 (4.46); mean change from baseline (SD) : -0.09 (4.74)  Significant effects for time (F (1,61) = 7.02, p <. 01, ηP2 =.104. Results also showed significant interaction effects between time and group. (F (1,61) = 7.92, p <.001, ηP2 =.116). | HADS, mean (SD), pre-treatment, 90 days:  EMDR-IGTP-OTS: 11.50 (4.53); 7.80 (5.42); mean change from baseline (SD) : 3.70 (5.50)  No treatment: 10.75 (4.05); 10.57 (5.30); mean change from baseline (SD) : 0.18 (5.23)  Significant effect for time (F (1, 61) = 7.46, p <.001, ηP2 =.109) and a significant interaction effect between time and group (F (1, 61) = 6.13, p<.01, ηP2=.091). |
| **Children, prevention, wait list/usual care, delayed** | | | | |
| Meentken 2020, 2021 | EMDR (37) vs WL (37) | 8 weeks, 8 months | Children’s Depression Inventory-2 (CDI-2), mean (SD) change from baseline  EMDR:  8 weeks: Child: 5.06 (6.23); Parent: 5.53 (6.83);  8 months: Child: 5.05 (6.21); Parent: 5.42 (7.22)  WL:  8 weeks: Child: 1.96 (7.08); Parent: 2.51 (7.59);  8 months: Child: 2.45 (6.90); Parent: 3.02 (7.43)  B (Uncorrected interaction of time × group), p, effect size:  8 weeks: Child −2.473, p=0.037, −.40; Parent: −2.551 , p=0.050, −.39;  8 months: Child -0.05, p=0.06, -0.25 (-0.73, 0.23); Parent: -0.00, p=0.95, -0.01 (-0.47, 0.45) | Screen for Child Anxiety Related Emotional Disorders (SCARED)-NL, mean (SD) change from baseline*  EMDR:  8 weeks: Child: 17.36 (22.4); Parent: 11.58 (16.95)  WL:  8 weeks: Child: 10.28 (21.11); Parent: 7.06 (22.61)  B (Uncorrected interaction of time × group.), p, effect size:  8 weeks: Child −6.834, p=0.101 ,−.34; Parent: −3.833, p=0.288, −.20 |
| Osorio, 2018 | EMDR-IGTP-OTS (12) vs no treatment (11) | 90 days | HADS, mean (SD), pre-treatment, 90 days:  EMDR-IGTP-OTS: 5.45 (3.13); 2.09 (2.50); mean change from baseline (SD) : 3.36 (3.13)  No treatment: 4.33 (3.98); 5.41 (4.87); mean change from baseline (SD) : -1.08 (4.90)  Significant interaction effects between time and group. (F (2, 42) = 6.49, p <.005,ηP  2= .236). | HADS, mean (SD), pre-treatment, 90 days:  EMDR-IGTP-OTS: 7.18 (3.99); 2.81 (1.77); mean change from baseline (SD) : 4.37 (3.66)  No treatment: 6.75 (5.01); 5.25 (4.57); mean change from baseline (SD) : 1.50 (5.26)  Significant effect for group (F (2, 42) =  10.64, p <.001, ηP2 = .336) and a significant interaction effect between time and group (F (2, 42) = 19.77, p <.05,ηP2= .163). |

** TAU: psychological support, oriented to life plan and emotions management

Table S4 Quality of life, functioning and others

| **Author, year**  **(study name)** | **Intervention (n)** | **Follow-ups** | **Quality of life** | **Functioning or dissociative experience** |
| --- | --- | --- | --- | --- |
| Jaberghaderi, 2019 | EMDR (40) vs TF-CBT (40) | 2 weeks (post treatment) | NR | NR |
| Banoglu, 2021 | EMDR-GP/C (47) vs WL (47) | Unclear | NR | World Health Organisation (WHO)-5 well-being index, Mean (SD), adjusted mean (SE)  EMDR: Baseline: 17.05, (5.25); Follow-up: 20.05 (SE=0.71)  WL: Baseline: 18.47 (5.25); Follow-up: 16.78 (SE=1.06)  Significant effect of EMDR on well-being scores (F(1,58)= 6.58, p= .013, hp 2= .10), indicating higher well-being score for EMDR group (Mean = 20.05, SE= .71, 95% CI [18.63, 21.47]) than for WL group (M=16.78, SE= 1.06, 95% CI [14.67, 18.89]). |
| Jaberghaderi, 2019 | EMDR (40) vs WL (59) | 2 weeks (post treatment) | NR | NR |
| Jimenez, 2020 | EMDR-PRECI (16) vs TAU** (16) | 90 days | NR | NR |
| Karadag, 2021 | EMDR (booklet) (88) vs WL (90) | 4 weeks | NR | NR |
| Molero, 2019 | EMDR-IGTP-OTS (93) vs no treatment (91) | 90 days | NR | NR |
| Meentken 2020, 2021 | EMDR (37) vs WL (37) | 8 weeks | Questionnaires for Children’s Health-Related Quality of Life (TACQOL): There were no significant difference between groups; both groups showed improvements on the secondary outcomes over time: HrQoL-body (b=0.07, P = 0.000), HrQoLMotor (b=0.03, P = 0.022), HrQoL-Autonomy (b=0.02, P = 0.034), HrQoL-Cognitive (b= 0.04, P = 0.004), HrQoL-Positive  Emotions (b= 0.03, P = 0.000), and HrQoL-Negative Emotions (b=0.02, P = 0.004); child-reported HrQoL-Social (b= 0.03, P = 0.053). | NR |
| Osorio, 2018 | EMDR-IGTP-OTS (12) vs no treatment (11) | 90 days | NR | NR |
